# Femtosecond Laser Mass Spectrometry and High Harmonic Spectroscopy of Xylene Isomers

**DOI:** 10.1038/s41598-018-22055-9

**Published:** 2018-02-28

**Authors:** Abdullah Alharbi, Andrey E. Boguslavskiy, Dane Austin, Nicolas Thiré, D. Wood, P. Hawkins, Felicity McGrath, A. S. Johnson, I. Lopez-Quintas, Bruno Schmidt, Francois Légaré, J. P. Marangos, Anh-Thu Le, Ravi Bhardwaj

**Affiliations:** 1 Department of Physics, Advanced Research Complex, University of Ottawa, 25 Templeton Street, Ottawa K1N6N5 Ontario, Canada; 2 King Abdulaziz City for Science and Technology (KACST), P.O. Box 6086, Riyadh, 11442 Saudi Arabia; 3 Blackett Laboratory, Imperial College London, London, UK; 4 Instituto de Química Física Rocasolano, IQFR-CSIC, Serrano 119, 28006 Madrid, Spain; 5 INRS-EMT, Advanced Laser Light Source, 1650 Lionel-Boulet Bvd, Varennes, J3X1S2 Canada; 6 J. R. Macdonald Laboratory, Physics Department, Kansas State University, Manhattan, Kansas 66506-2604 USA

## Abstract

Structural isomers, molecules having the same chemical formula but with atoms bonded in different order, are hard to identify using conventional spectroscopy and mass spectrometry. They exhibit virtually indistinguishable mass spectra when ionized by electrons. Laser mass spectrometry based on photoionization of the isomers has emerged as a promising alternative but requires shaped ultrafast laser pulses. Here we use transform limited femtosecond pulses to distinguish the isomers using two methods. First, we probe doubly charged parent ions with circularly polarized light. We show that the yield of doubly charged ortho-xylene decreases while para-xylene increases over a range of laser intensities when the laser polarization is changed from linear to circular. Second, we probe high harmonic generation from randomly oriented isomer molecules subjected to an intense laser field. We show that the yield of high-order harmonics varies with the positioning of the methyl group in xylene isomers (ortho-, para- and meta-) and is due to differences in the strength of tunnel ionization and the overlap between the angular peaks of ionization and photo-recombination.

## Introduction

A key task in analytical chemistry is to identify isomers – molecules that have the same chemical formula but have different chemical properties and reactivities. This is in part due to their critical role in pharmacology and medicinal chemistry^1–3^. Isomeric identification techniques are either spectroscopic or spectrometric. Spectroscopy exploits specific transitions induced in isomers by electromagnetic radiation. UV-visible spectroscopy relies on electronic transitions to provide information about *π*-bonds and conjugated systems^4^, while Raman and Infrared spectroscopies use molecular vibrations to provide information on functional groups^5,6^. Nuclear magnetic resonance depends on chemical shift to map carbon-hydrogen framework^7,8^.

Mass spectrometry is an alternate analytical method with very low detection limit and high sensitivity. As a result, it is widely used in applications ranging from toxicology and biomedical research to forensics and environmental research^9–16^. However, conventional mass spectrometers, with electron impact ionization sources, provide accurate compositional information but molecular recognition is hard since they produce nearly identical fragmentation patterns. Therefore, mass spectrometry is often combined with gas/liquid chromatography, ion-molecule collisions, chemical reactivity and ion traps^17–22^. To enhance molecular recognition, lasers are used in mass spectrometry to control ionization and fragmentation. For example, in resonance enhanced multiphoton ionization isomer selectivity is achieved by exploiting differences in the dynamics of intermediate states excited by a tunable laser followed by ionization^23–25^.

Femtosecond lasers offer a new dimension to mass spectrometry with their ability to control a wide range of pulse characteristics and thereby chemical reactions^26,27^. Pulse shaping was recently demonstrated as a viable method to identify isomers using a benchmark system, xylene isomers, through their fragmentation fingerprints. In one approach, different relative ion yields of the major fragment (C_7_H_7_^+^) ions were produced by dividing the frequency spectrum of the femtosecond pulse into different groups and changing their phase retardation^28^. An alternate approach exploited the time domain of the pulse, instead of the frequency domain^29^. By varying the chirp of the laser pulse, differences in the ion yields of the two isomers, in particular the small fragments, were amplified. However, use of fragmentation with tailored optical pulses makes it difficult to understand the underlying physics because of insufficient information on vibrational manifold of complex molecules and energy redistribution into multitude of modes.

In this article, we demonstrate differentiation of structural isomers with simple transform-limited femtosecond pulses using ionization as a probe. We use xylene isomers to test new femtosecond laser-based spectrometric and spectroscopic methods. Both depend on the extent of overlap between the molecular ion and electron wave packet in a recollision process where the ionized electron is driven back after propagation in a linearly polarized laser field and can undergo inelastic scattering or recombination. In the spectrometric approach, turning off the recollision process by switching the laser polarization from linear to circular provides isomer selectivity between o- and p-xylene. A time-of-flight mass spectrometer was used to monitor doubly charged ion yields of the two isomers. In the spectroscopic approach, isomers are distinguished by monitoring the emission of light (instead of molecular ions and fragments) through the generation of high-order harmonics (HHG)^30^ produced when an ionized electron returns to the parent ion and undergoes recombination. The kinetic energy of the recolliding electron during the recombination process is converted to photons that carry information on the molecular structure and dynamics. Differences in the position of a functional group in structural isomers such as xylene would likely be reflected in the angle dependence of the ionization and recombination processes. As a result, distinctly different harmonic spectra can be observed. Since the ionization and the recombination are coupled in a HHG process at the sub-cycle level, such differences can exist even in randomly oriented stereoisomers, that were observed experimentally^31–33^. We report differences in both the harmonic yields and on the polarization dependence of HHG.

## Experimental Results

### Femtosecond laser mass spectrometry – probing di-cations of xylene isomers

**Figure 1:**
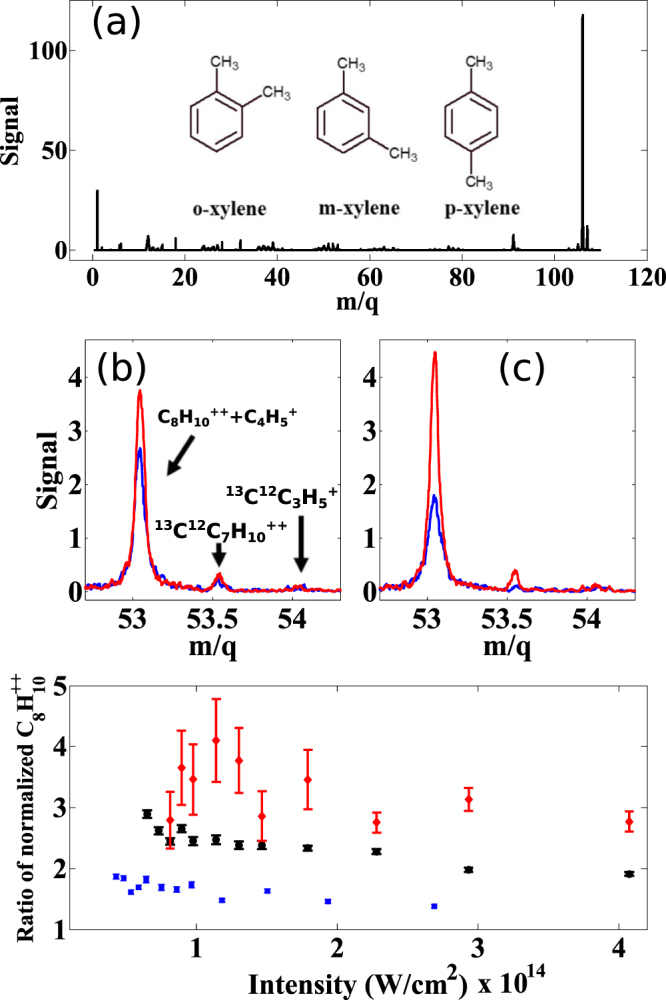
Femtosecond laser mass spectrum of o- and p-xylene. (**a**) Time-of-flight mass spectrum of o-xylene at 800 nm and laser intensity of 10^14^/W/cm^2^. The inset shows the structure of the three xylene isomers. (**b,c**) Portion of the mass spectrum containing the signal of doubly charged ions of p-xylene (red) and o-xylene (blue) for linear polarization and, (**c**) for circular polarization. Only the portion of spectra containing the signals of doubly charged ions are shown. (**d**) The ratio of doubly charged p-xylene signal at m/q = 53 to that of o-xylene at 800 nm for linear polarization (blue squares) and circular polarization (black circles). Also is shown the same ratio for the isotope signal at m/q = 53.5 for circularly polarized light (red diamonds). The doubly charged signal was normalized to the parent ion.

The three xylene isomers differ in the carbon atom positions on the benzene ring to which the two methyl groups are attached, as shown in the schematic in the inset of Fig. 1a. The main part of Fig. 1a shows the mass spectrum obtained in o-xylene using a transform limited pulse with intensity 10^14^ W/cm^2^. The spectrum was nearly identical for linear and circular polarization, and also was unchanged upon changing the sample to p-xylene. The absolute ion yield of singly charged o-xylene (obtained by integrating the peak at *m/q* = 106) is slightly higher than p-xylene in the intensity range of 2–30 × 10^13^ W/cm^2^ for both laser polarizations (see Supplementary Figure S1). This is in good agreement with the numerical simulation of the total ionization probability (see Fig. 4b,c). However, these small differences in the absolute ion yields does not allow unambiguous differentiation of the two isomers.

We now show that monitoring the doubly charged parent ion yield enables better differentiation between o- and p-xylene than monitoring singly charged parent and fragment ions. Figure 1(b,c) shows low *m/q* portion of the mass spectra corresponding to doubly charged o- and p-xylenes for linear and circular polarization, respectively. The peak at *m/q* = 53 corresponds to doubly charged parent ion with some contribution from C_4_H_5_ fragment. The peak at 53.5 corresponds to the doubly charged molecular isotope ^13^C^12^C_7_H_10_. Comparison of the measured isotopomer ratio with known value of 8.9% suggests there is less than 25% contribution of the molecular fragment at *m/q* = 53 for both linearly and circularly polarized light.

Two key observations can be made from Fig. 1(b,c). First, the doubly charged parent ion yield is enhanced in p-xylene despite the fact that the singly charged ion yield is lower than in o-xylene. Second, when the polarization is changed from linear to circular the doubly charged parent ion yield is reduced in o-xylene whereas it remained the same or slightly increased in p-xylene. This behaviour is persistent over an intensity range of 2–20 × 10^13^ W/cm^2^ (see Supplementary Figure S2). There are two mechanisms that contribute to the doubly charged ion signals: the so-called sequential and non-sequential. The latter is caused by inelastic collision of the returning electron with the parent ion (similar to e-2e process in the laser-free counterpart) and therefore can easily be manipulated by changing the laser polarization. In fact, it is well-known that the non-sequential mechanism is exponentially suppressed with increased driving laser’s ellipticity, since the continuum electron is driven further away from the parent ion. For a circularly polarized pulse, contribution from non-sequential double ionization essentially vanishes. The results shown in Fig. 1(b,c) clearly suggest much larger contribution from non-sequential double ionization in o-xylene, as compared to p-xylene. Also, for circular polarization the contribution of sequential double ionization in p-xylene is higher than o-xylene.

The different polarization dependences of the two isomers enables distinguishing them by using transform-limited pulses. Figure 1d shows the ratio of the doubly charged ion yields of p-xylene to that of o-xylene, normalized to their respective parent ions, for linear (squares) and circular (circles) polarizations at different intensities. For linear polarization, the ratio is ~1.5 but is enhanced for circular polarization. The differences are more pronounced when isotopes are considered (open circles in Fig. 1d) due to the absence of fragment contribution. These results are unaffected even after the intensity scaling to ensure relative ion yields for different laser polarizations are identical^34^ (see Supplementary material).

### High harmonic generation in xylene isomers

**Figure 2:**
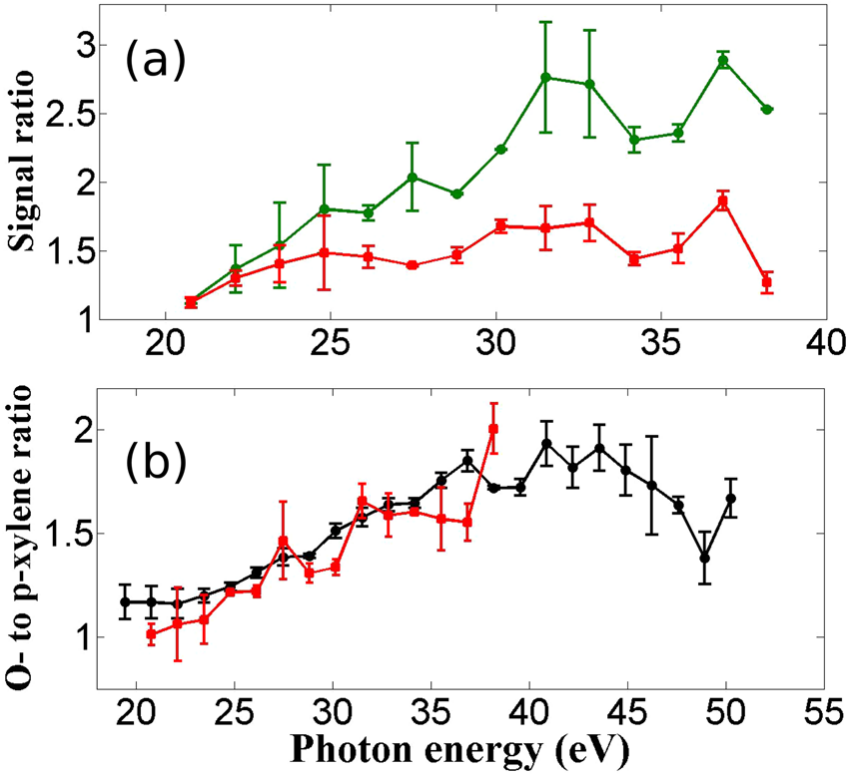
HHG yields in Xylenes. (**a**) Ratio of HHG yields in o-xylene(green circles) and p-xylene (red sqaures) with respect to m-xylene at 1850nm and an intensity of 6.5 × 10^13^ W/cm^2^. (**b**) HHG yields ratio of o-xylene to p-xylene at 1850nm for two laser intensities 6.5 × 10^13^ W/cm^2^ (red squares) and 1.1 × 10^14^ W/cm^2^ (black circles).

Figure 2a shows the relative HHG yield for o-xylene (green) and p-xylene (black) with respect to m-xylene at an intensity of 6.5 × 10^13^ W/cm^2^ and a wavelength of 1850 nm. The longer wavelength ensures (i) adiabatic ionization in molecules with low ionization energy, and (ii) extended range of harmonics up to 50 eV that is essential for isomer identification.

Two key observations can be made from Fig. 2a. First, the harmonic yields from m-xylene are always lower than o- and p-xylene (resulting in ratios that are greater than one). The results suggest that o-xylene can be distinguished from p- and m-xylenes. Although the differences between p-xylene and m-xylene are small, they are measurable and present even at shorter wavelengths (see Supplementary Figure S4). Second, harmonic yield from o-xylene is higher than p-xylene and this difference increases progressively from low to high-order harmonics. Figure 2b shows that this behaviour is the same at laser intensities of 6.5 × 10^13^ W/cm^2^ (red) and 1.1 × 10^14^ W/cm^2^ (black). The distinction between the two isomers differs by a factor of 1.5 to 2 for higher-order harmonics. However, at 1430 nm, the ratio of o-xylene to p-xylene is close to unity (not shown); it therefore makes it more difficult to differentiate the two isomers at 1430 nm compared to the longer wavelength.

### HHG with elliptically polarized light

**Figure 3:**
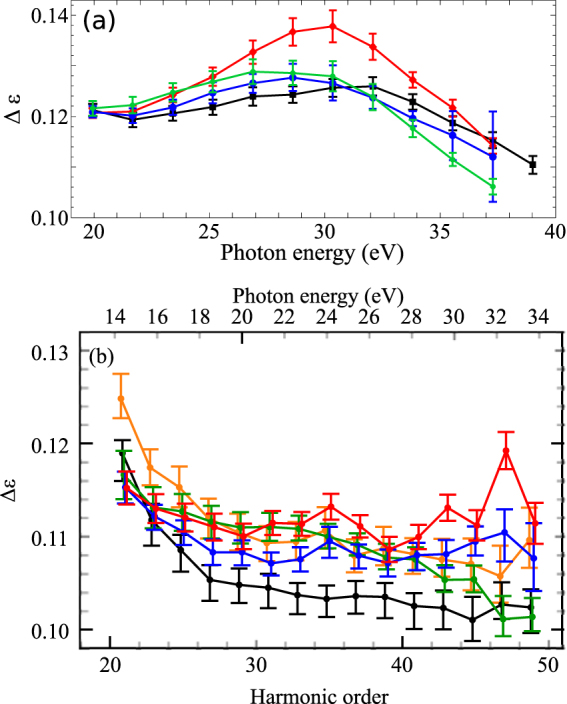
Ellipticity width dependence on harmonic order. (**a**) The ellipticity width ${\rm{\Delta }}\epsilon$ of harmonics as a function of photon energy for benzene (black), p-xylene (green), o-xylene (blue) and m-xylene (red) at a laser intensity of 6 × 10^13^ W/cm^2^ and wavelength of 1430 nm. (**b**) The same measurement but using a wavelength of 1800 nm and an intensity of 4 × 10^13^ W/cm^2^. Also shown is ${\rm{\Delta }}\epsilon$ for toluene (orange). ${\rm{\Delta }}\epsilon$ is the half width at half maximum and was obtained from the Gaussian fit to the experimental data as a function of ellipticity for each harmonic. Measurements at1430 nm and 1850 nm were carried out ALLS (Montreal, Canada) and Imperial College (London, UK), respectively.

Probing the dependence of high harmonics on the ellipticity of the driving laser field, defined as the ratio between the minor and major components of the driving laser field, provides insight into the transverse spreading of the electron wavefunction after tunnelling and the molecular structure. Figure 3 shows the variation of half width at half maximum, ${\rm{\Delta }}\epsilon$, in xylenes at 1430 nm and 1800 nm. ${\rm{\Delta }}\epsilon$ was obtained from the Gaussian fit to the experimental data as a function of ellipticity for each harmonic. The results were compared to benzene, since it is the building block of xylene isomers with two additional methyl groups attached to the benzene ring in different positions. The errors represent 95% confidence interval of the Gaussian fits.

Larger ${\rm{\Delta }}\epsilon$ generally indicates wider spreading of the electron wavefunction. In atoms, within the framework of the three-step model, the quantum diffusion of the electron wavefunction causes ${\rm{\Delta }}\epsilon$ to decrease (increase) monotonically for short (long) trajectories with the increasing harmonic order. Under our experimental conditions where short trajectories were chosen by appropriate phase matching, ellipticity dependence of Xe exhibited (not shown) a monotonic decrease in ${\rm{\Delta }}\epsilon$ with harmonic order.

At 1430 nm (Fig. 3a), the electron wave packet spreading after tunnel ionization is largest in m-xylene compared to the other molecules resulting in weaker ellipticity dependence. There are two noticeable features: (i) Ellipticity dependence is relatively strong (${\rm{\Delta }}\epsilon$ is small) for lower and cutoff harmonics and is weak around the 35^*th*^ harmonic corresponding to a photon energy of 30.3 eV. (ii) Around 30 eV, p- and o- xylenes undergo a transition from weaker to stronger ellipticity dependence relative to benzene. There also appears to be a similar transition between p- and o-xylenes with ${\rm{\Delta }}\epsilon$ being larger (lower) in p-xylene below (above) 32 eV. These observations reflect the complexity of the HHG process in complex molecules.

At 1800 nm (Fig. 3b), the weaker ellipticity dependence of m-xylene at high orders is reproduced. ${\rm{\Delta }}\epsilon$ is ≈ 15% larger at 1430 nm than at 1800 nm. Tunnelling and wavepacket spreading considerations in atoms^35^ predict ${\rm{\Delta }}\epsilon$ ∝ *λ*^−1^*I*^−1/4^ i.e. 14% larger at 1430 nm when considering the slightly different intensities used in the two experiments. Our measurements are therefore in good agreement with this prediction. There are two notable differences between the two wavelengths. (1) At 1800 nm, ${\rm{\Delta }}\epsilon$ decreases rapidly over harmonic 21–29 (or photon energies of 14–20 eV) and then flattens out a little. However, this range of energy was not recorded in the 1430 nm measurement. (2) The transition that occurs at harmonic 35 at 1430 nm where the ${\rm{\Delta }}\epsilon$ of benzene goes above that of o- and p-xylene does not occur at 1800 nm. However, there seems to be a transition in ${\rm{\Delta }}\epsilon$ between p- and o-xylene around 26 eV similar to 1430 nm data. Finally, we note that toluene, which was not measured at 1430 nm, exhibits similar ellipticity dependence to the xylenes except below 20 eV, where its ${\rm{\Delta }}\epsilon$ is slightly larger. These results indicate that while HHG spectroscopy with laser polarization can be used to distinguish m-xylene from other isomers, the method can be quite sensitive to the laser wavelength. The origin of this sensitivity is not known at present.

## Theoretical results

In this section we present theoretical simulations for HHG with linearly polarized pulses and single ionization from xylenes. Theoretical treatments for ellipticity dependence of HHG and double ionization are much more complicated, so we leave them for a future work. For a given molecular alignment with respect to the laser polarization, the induced dipole of HHG is proportional to the product of tunneling ionization amplitude and photo-recombination dipole^36,37^. We therefore analyze angular dependence of both factors. Generally, one would expect the strongest HHG yield originated from the largest angular overlap of the two factors. Detailed analysis below indeed reveals the nature of the experimentally observed large ratios of o- and p-xylene with respect to m-xylene as mainly due to this overlap. This is in contrast to the case of 1,2-dichloroethylene (DCE) stereoisomers, in which the strong differences between the HHG yields from cis- and trans-DCE were attributed mainly to the destructive interference from different molecular alignments in trans-DCE^32^.

### Angle dependent tunneling ionization

**Figure 4:**
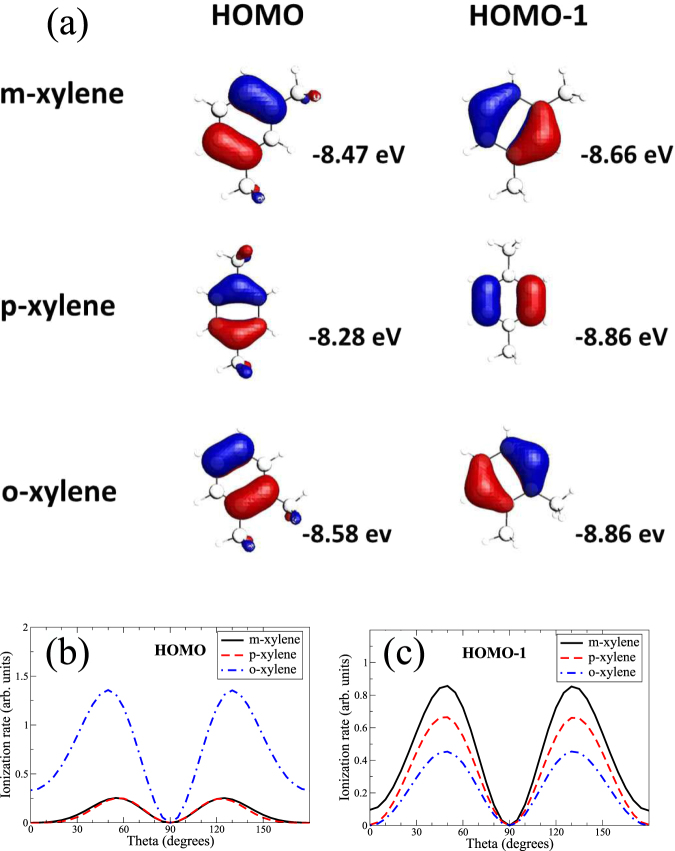
Angle dependent ionization yields. (**a**) Molecular orbitals with corresponding ionization energies for xylene isomers. (**b**) Integrated yield vs polar angle from HOMO and (**c**) from HOMO-1, respectively, for each isomer. Results only include electron emission along the laser polarization direction. Laser wavelength and intensity used in the simulations were 1850 nm and 0.8 × 10^14^ W/cm^2^, respectively.

Figure 4a shows the two highest occupied molecular orbitals and ionization energies for the three xylene isomers. For m- and o-xylene, most of the electron density distribution is on the ring and their HOMO’s and HOMO-1’s have similar shape. In p-xylene, the distribution in the ring for HOMO is similar to the HOMO-1 of m- or o-xylene and vice-versa. Based on the small differences in ionization energy and the shape of these molecular orbitals, one would generally expect ionization from these isomers to be similar. Lower molecular orbitals were not considered as they are energetically well separated from the HOMO and HOMO-1.

**Figure 5:**
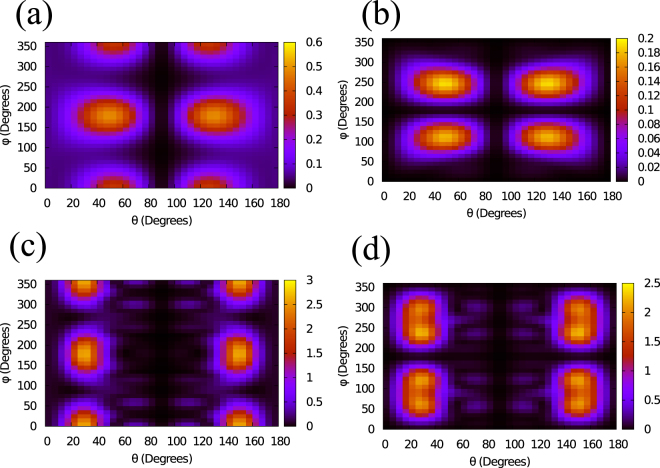
Tunneling ionization and photo-recombination cross sections. (**a**,**b**) Angle-dependent ionization from HOMO (left panel) and HOMO-1 (right panel), for o-xylene. Here the laser polarization direction is given by polar angle *θ* and azimuthal angle *ϕ*, defined in the molecular frame (see Supplementary). (**c,d**) O-xylene recombination cross section vs photon polarization direction at 36 eV for HOMO (left panel) and HOMO-1 (right panel). The electron emission is along polarization direction. Laser with wavelength of 1850 nm and an intensity of 0.8 × 10^14^ W/cm^2^ was used in simulations.

The calculations were performed using the Stark-corrected SFA^38,39^ with the laser intensity of 0.8 × 10^14^ W/cm^2^ and wavelength of 1850 nm. The full angle dependent ionization is shown in Fig. 5(a,b), for the HOMO and the HOMO-1 of o-xylene, respectively (for more details, see Supplementary Figure 5). Overall, the angular dependence reflects quite closely the shape of the molecular orbital for all cases. To have a more quantitative idea, we integrate the ionization yield over the azimuthal angle. The polar angular dependence of ionization for HOMO and HOMO-1 varies strongly, as shown in Fig. 4b and c. Nevertheless, the total (angle-integrated from both HOMO and HOMO-1) ionization yields for xylene isomers differ from one another only by about 20%. This is in good agreement with the experimental finding (see Supplementary Figure 1).

Ionization from o-xylene is strongest with a dominant contribution from the HOMO. Ionization from m-xylene is slightly stronger than p-xylene. For both these isomers HOMO-1 yields are stronger than the HOMO. The polar angular dependence has a peak near *θ* = 50° (and 130°), except for the HOMO of p- and m-xylenes where it is near *θ* = 60° (and 120°). These results have strong consequences on the HHG yield, as shown below.

### Photo-recombination cross sections

Photo-recombination (time-reversal of photo-ionization) was calculated using ePolyScat package^40,41^. Figure 5(c) and (d) show the differential cross section for the electron momentum along the photon polarization for o-xylene at photon energy of 36 eV for HOMO and HOMO-1, respectively. Again, for each of these molecular orbitals, the angular dependence resembles quite closely the shape of its electron density, although some shift in the peak position along *θ* can be seen, as compared to the tunneling ionization, shown in Fig. 5(a) and (b). In particular, the cross section has a peak near *θ* = 30° (and 150°). Similar close resemblance was found for the other isomers as well. Furthermore, the cross sections for all three isomers were found to be rather similar in magnitude for any fixed energy below about 50 eV.

Based on the above findings, the main factors that distinguish the HHG yields in xylene isomers are the tunneling ionization rate and the extent of its overlap with recombination. For the HOMO, o-xylene not only has the strongest tunnel ionization, but it also has strong overlap between ionization and recombination, while the other two isomers have weaker ionization as well as weaker overlap since their tunnelling ionization peaks move further away from the photo-recombination peak position. Therefore, in p- and m-xylenes, the dominant contributions are expected to be from the HOMO-1.

### HHG simulations

**Figure 6:**
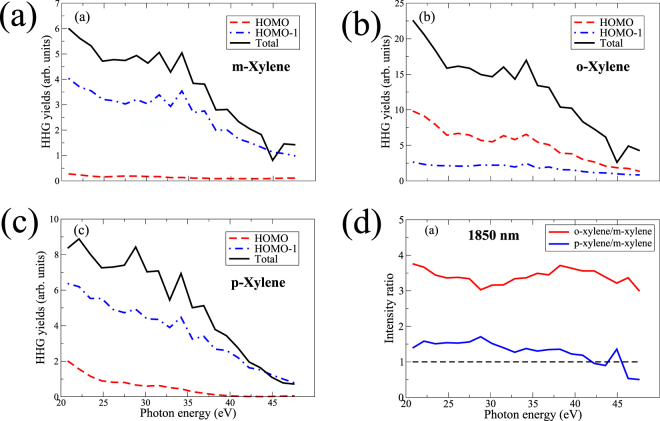
Calculated high harmonic spectra of xylenes. for HOMO (red), HOMO-1 (blue) and total signal (black) for (**a**) m-xylene, (**b**) o-xylene, and (**c**) p-xylene. (**d**) Calculated harmonic ratios of p-xylene to m-xylene (blue) and o-xylene to m-xylene (red). A 10-cycle pulse with wavelength of 1850 nm and intensity of 0.8 × 10^14^ W/cm^2^ was used in simulations.

The above theoretical results can be summed up in Fig. 6a–c showing high harmonic spectra of xylene isomers, calculated using the quantitative rescattering (QRS) theory^36,37,42^. To mimic the effect of macroscopic propagation, the HHG results were obtained with laser intensity averaging^36,42^. No depletion effect was taken into account in our calculations. HHG yield from HOMO in o-xylene (Fig. 6b) is much stronger than that from the other two isomers (Fig. 6a,c). The results also reveal that for m-xylene and p-xylene, HHG from the HOMO-1 actually dominates HHG from the HOMO. Also, HHG yield is stronger in p-xylene as compared to m-xylene, mostly due to a stronger overlap between tunnel ionization and photo-recombination in p-xylene, although ionization is slightly stronger in m-xylene. Total HHG induced dipole for each isomer is obtained by a coherent sum of contributions from the HOMO and HOMO-1. We found that the two contributions are mostly in phase for all isomers, see Fig. 6a–c.

Figure 6d shows the calculated ratio of o-xylene (p-xylene) to m-xylene in red (blue). These results reproduce qualitatively the experimentally obtained ratios at 1850 nm. We also found that these ratios remain nearly unchanged at the lower intensity of 0.4 × 10^14^ W/cm^2^. The theoretical ratios at laser wavelength of 1430 nm with intensity of 0.6 × 10^14^ W/cm^2^ remain nearly the same as for 1850 nm case (see Supplementary Figure S6.) whereas the experimental results show a stronger dependence on the laser wavelength (see Supplementary Figure S4). A possible reason is that the ionization calculation based on the SFA does not fully account for the laser wavelength dependence, especially since it neglects all excited states of the targets.

## Discussion

HHG spectroscopy is well known for its inherent spatial and temporal resolution that enabled to image orbitals and probe dynamics with Angstrom and attosecond precision^43–46^. Our results show that it is also highly sensitive to small changes in the molecular environment^47^. The subtle differences exhibited by xylene isomers both in the high harmonic yield and their dependence on the laser polarization lays the foundation for future experiments on time-resolved dynamics of ring opening and ring permutation processes exhibited by these molecules. For example, m-xylene is known to undergo ring permutation upon photoexcitation by UV light to form o-xylene through an intermediate state^48^. The inherent spatial and temporal resolution offered by HHG spectroscopy combined with its ability to distinguish the isomers can enable to track such interesting isomerization dynamics.

Both of our approaches to identify molecular isomers (probing double ionization and high harmonic spectroscopy) do not, in general, require polarization shaping. Linearly polarized transform limited light pulses are sufficient to differentiate xylene isomers without the need for any pulse shaping either in the frequency- or time-domain. However, the sensitivity can be further enhanced in the spectrometric technique by switching laser polarization from linear to circular. Therefore, it is a direct, simpler and faster method than the existing femtosecond laser mass spectrometry and other conventional tandem mass spectrometric techniques.

## Methods

### Materials

Xylenes, obtained from Sigma-Aldrich in a liquid form with 97% purity, were introduced into the vacuum system by exploiting their high vapour pressure. In the interaction region, the molecules were randomly oriented.

### Femtosecond laser mass spectrometry

Ionization experiments were conducted using a time-of-flight mass spectrometer in Wiley-McClaren configuration with a 30 cm field-free flight tube. A Ti: Sapphire laser (800 nm, 40 fs pulses, operating at 1 kHz) was used to ionize xylenes introduced, at a pressure of 10^−7^ torr, into the vacuum system with a base pressure of ~10^−9^ torr. The ions were detected by a microchannel plate (MCP). The mass spectrum was calibrated using Xe ions. Laser intensity was calibrated by measuring the saturation intensity of Ar. The ion yield as a function of laser pulse energy was plotted in a semi-log plot. Extrapolation of the linear portion of the ion signal curve defined the saturation energy, and comparison with the calculated ion yield using the ADK model provided the saturation intensity.

### High harmonic generation at ALLS

A finite gas cell was used to generate harmonic emission. Experiments were conducted at the Advanced Laser Light Source (ALLS) facility in Montreal, Canada. An optical parametric amplifier (OPA) pumped by 5 mJ, 800 nm, 30 fs pulses from a Ti: Sapphire laser operating at 100 Hz, produced energetic mid infrared pulses in the wavelength range of 1400–1850 nm. Harmonics were generated in by focusing mid infrared pulses from OPA with a 50 cm CaF_2_ lens into the gas cell. The gas cell consisted of two concentric cylindrical cells, each having two in-line pinholes of 600 *μ*m. Gas was delivered to the inner cell through a 1/4-inch Teflon tube connected to a vial with liquid xylenes after multiple freeze-thaw cycle to remove background molecules. The pressure in the gas cell was monitored with a Baratron gauge. The outer cell was connected to a high throughput scroll pump. The gas cell has an effective interaction length of 10 mm and is mounted on an XYZ manipulator to permit precision alignment with respect to the laser axis.

The source and detector chambers were differentially pumped through a 3 mm tube. Different harmonic orders are detected using a grazing-incidence concave grating which disperses and focuses them onto a MCP coupled to a phosphor screen. The screen was imaged with a charge coupled device (CCD) camera. The MCP voltage was adjusted to keep the detection in the linear regime. The harmonic spectra were obtained under optimal phase-matching conditions by adjusting the position of the laser focus with respect to the pinholes and the absolute pressure inside the gas cell. The spectrometer was calibrated by using the Cooper minimum in the HHG spectrum of argon as a reference. This technique was validated in the past by measuring the harmonic spectrum with an aluminium filter inserted in the beam path and comparing it with the calculated transmission spectrum of a known thickness of aluminium. Laser intensity was calibrated by monitoring cut-off harmonics in Ar and validated in the past by measuring its saturation intensity using a fast ionization gauge by monitoring the ion signal. A combination of half wavelplate (HWP) and a polarizer were used to vary the laser intensity. The ellipticity of the incident light (defined as the ratio of the two orthogonal electric fields of the laser light) was controlled by a combination of another adjustable HWP and a fixed quarter-wave plate. This arrangement ensured that the main axis of the ellipse is fixed in space.

### High harmonic generation at Imperial College

The harmonics were generated in a continuous flow jet formed from a nozzle of diameter 0.2 mm that was backed by ≈0.3 bar xylene vapour. Experiments were conducted by focusing mid-infrared pulses from the idler of an OPA (TOPAS HE, Lightconversion) centred at 1800 nm with a 50 cm CaF_2_ lens into the 0.5 mm diameter gas jet. The OPA was pumped by 8 mJ, 800 nm, 25 fs pulses from a Ti:Sapphire laser operating (Red Dragon, KM Labs) at 1 kHz. The OPA beam was passed through an evacuated 400 *μ*m diameter hollow capillary acting as a spatial filter that ensured 0.6 mJ pulses of 50 fs duration (confirmed by FROG measurement) in a HE_11_ mode were delivered to the experiment. The focal spot was measured to have *e*^−2^ radius 100 m, so the Rayleigh range was 17 mm and the generation occurred in the loose focusing limit. The HHG detection system was composed of a flat-field concave grating and a microchannel plate detector coupled to a phosphor screen and charge-coupled device (CCD) camera. The harmonics were produced in a non-saturated regime, confirmed by checking linear scaling of HHG cut-off with intensity, and for these measurements an intensity of around 3 × 10^14^ Wcm^−2^ was used. The ellipticity dependence was obtained by measuring the harmonics while rotating a quarter wave plate to 26 angles over a 36° range in random order. Reference measurements at linear polarization were interlaced to correct for laser drift. Error bars were obtained by bootstrapping with multiple angle scans (typically 3).

### Molecular orbitals and ionization energies

Xylene isomers are substituted benzene derivatives by two methyl groups. This substitution removes the degeneracy of the two *π* HOMO orbitals of benzene and somewhat lowers the ionization energy (*I*_*p*_ of benzene = 9.25 eV). The molecular orbitals and ionization energies for the three xylene isomers were obtained by Gaussian quantum chemistry code^49^. The ionization energies of m-, p- and o-xylene were 8.47, 8.28 and 8.58 eV, respectively. This is in close agreement with experiment values of 8.56, 8.44 and 8.56 eV for m-, p-, and o-xylene, respectively, obtained by photoelectron spectroscopy.

### Calculation of ionization rates

Angle dependent ionization rates were carried out using the strong-field approximation (SFA)^50,51^ within the single-active electron approximation. We used the wavefunctions generated from the Gaussian quantum chemistry code^49^ at the Hartree-Fock level with the augmented correlation-consistent polarized valence triple-zeta (aug-cc-pVTZ) basis set. Since the HOMO and HOMO-1 have quite close ionization energies, well separated from lower molecular orbitals, we only account for these two orbitals in our simulations. Ionization yields from the HOMO and HOMO-1 were added up to obtain the total yield for each isomer, resulting in the ratios of 1:0.85:0.8 for o-xylene:m-xylene:p-xylene. The calculation were performed with a 3-cycle pulse with the laser intensity of 0.8 × 10^14^ W/cm^2^ and wavelength of 1850 nm. Here we have included the Stark shift correction for the polar molecules (o-xylene and m-xylene), following refs^38,39^. The ratios are virtually unchanged with a half-cycle laser calculation. They only slightly change without the Stark shift correction.

### Calculation of high harmonic spectra

High harmonic spectra of xylene molecules were calculated within a single-molecule response approximation using the quantitative rescattering (QRS) theory^36,37,42^. Within the QRS, HHG yield for a fixed photon energy and fixed laser direction (or molecular alignment) is proportional to the product of tunneling ionization rate and (differential) photo-recombination cross sections. To compare with experiments, averaging over isotropic molecular alignment distribution was carried out. The actual QRS calculations are done at the level of complex amplitudes, although it was found for xylenes that qualitative understanding of the relative HHG intensities can be gained even without the phase consideration. The calculation of tunneling ionization was described above. For HHG simulations, in order to account for a definite sub-cycle electric field direction, only a half-cycle at the peak of the laser pulse is allowed to ionize the molecules. Furthermore, only electron emission along the laser direction is taken into account. The photo-recombination dipoles were calculated using ePolyScat package^40,41^. Total HHG induced dipole for each isomer is obtained by a coherent sum of contributions from the HOMO and HOMO-1. To mimic the effect of macroscopic propagation, the induced dipoles were averaged over a range of ±10% of the mean laser intensity.

## Electronic supplementary material

supplementary information

## References

[1] Chhabra N, Aseri ML, Padmanabhan D (2013). A review of drug isomerism and its significance. Int. J. App. Basic Med. Res..

[2] Nguyen LA, He H, Pham-Huy C (2006). Chiral Drugs: An Overview. Int. J. Biomed. Sci..

[3] Davies NM, Teng XW (2003). Importance of Chirality in Drug Therapy and Pharmacy Practice: Implications forPsychiatry. Advances in Pharmacy.

[4] Lambert JB, Gronert S, Shurvell HF, Lightner D, Cooks RG (2011). Organic Structural Spectroscopy.

[5] Lin-Vien D, Colthup NB, Fateley WG, Grasselli JG (1991). The Handbook of Infrared and Raman Characteristic Frequencies of Organic Molecules.

[6] Infrared and Raman spectroscopy: methods and applications, Ed. Schrader, B., VCH publishers, New York, 1995.

[7] Parker D (1991). NMR determination of enantiomeric purity. Chem. Rev..

[8] Marek R, Lycka A (2002). 15N NMR Spectroscopy in Structural Analysis. Current Organic Chemistry.

[9] Strathmann FG, Hoofnagle AN (2011). Current and Future Applications of Mass Spectrometry to the Clinical Laboratory. Journal of Clinical Pathology.

[10] Finehout EJ, Lee KH (2004). An introduction to mass spectrometry applications in biological research. Biochemistry and molecular biology Education.

[11] Smith, D. L. Mass Spectrometry Applications in ForensicScience, E*ncyclopedia of Analytical Chemistry*, 10.1002/9780470027318.a9121.

[12] Chen, C.-H., Mass Spectrometry for Forensic Applications, E*ncyclopedia of Analytical Chemistry*, 10.1002/9780470027318.a1115.

[13] Medved M (1991). Mass Spectrometry in Environmental Analysis. Rapid communications in mass spectrometry.

[14] Wang X, Wang S, Cai Z (2013). The latest developments and applications of mass spectrometry in food-safety and quality analysis. Trends in Analytical Chemistry.

[15] Munoz Llamas A, Bosch Ojeda C, Sanchez Rojas F (2007). Process Analytical Chemistry–Application of Mass Spectrometry in Environmental Analysis: An Overview. Applied Spectroscopy Reviews.

[16] Lebdev AT (2013). Environmental Mass Spectrometry. Annual Review of Analytical Chemistry.

[17] Sparkman, D. O., Penton, Z., & Kitson, F. G. Gas Chromatography and Mass Spectrometry: A Practical Guide, 2nd Ed., Academic Press 2011.

[18] Robert E. Ardrey, Liquid Chromatography – Mass Spectrometry: An Introduction, John Wiley & Sons, Ltd., 2003.

[19] Niessen, W. M. A. Liquid chromatography–mass spectrometry, (Taylor & Francis, 2007).

[20] Wu HF, Chuan YJ (2003). ., Isomer differentiation by combining gas chromatography, selective self-ion/molecule reactions and tandem mass spectrometry in an ion trap mass spectrometer. Rapid communications in mass spectrometry.

[21] Wu HF, Wu WF (2003). Comparing differentiation of xylene isomers by electronic ionization, chemical ionization and self-ion/molecule reactions and the first observation of methyne addition ions for xylene isomers in self-ion/molecule reactions for non-nitrogenated compounds. Rapid communications in mass spectrometry.

[22] Bjarnason A (1996). Xylene Isomer Mass Spectral Identification through Metal Ion Chemistry in an FTICR. Anal. Chem..

[23] Tembreull R, Lubman DM (1984). Use of resonant two-photon ionization with supersonic beam mass spectrometry in the discrimination of cresol isomers. Analytical Chemistry.

[24] Weickhardt C, Zimmermann R, Schramm KW, Boesl U, Schlag EW (1994). Laser mass spectrometry of the Di-, Tri- and tetrachlorobenzenes: Isomer-selective ionization and detection. Rapid Communications in Mass Spectrometry.

[25] Tembreull R, Sin CH, Li P, Pang HM, Lubman DM (1985). Applicability of resonant two-photon ionization in supersonic beam mass spectrometry to halogenated aromatic hydrocarbons. Analytical Chemistry.

[26] Assion A (1998). Control of Chemical Reactions by Feedback-Optimized Phase-Shaped Femtosecond Laser Pulses. Science.

[27] Levis RJ, Menkir GM, Rabitz H (2001). Selective bond dissociation and rearrangement with optimally tailored, strong-field laser pulses. Science.

[28] Dela Cruz JM, Lozovoy VV, Dantus M (2005). Quantitative mass spectrometric identification of isomers applying coherent laser control. The Journal of Physical Chemistry A.

[29] Urbasch G, Breunig HG, Weitzel K-M (2007). Distinction of ortho- and para-Xylene by Femtosecond-Laser Mass Spectrometry. ChemPhysChem.

[30] Corkum PB (1993). Plasma Perspective on Strong-Field Multiphoton Ionization. Phys. Rev. Lett..

[31] Wong MCH, Brichta J-P, Spanner M, Patchkovskii S, Bhardwaj VR (2011). High-harmonic spectroscopy of molecular isomers. Phys. Rev. A.

[32] Le AT, Lucchese RR, Lin CD (2013). High-order-harmonic generation from molecular isomers with midinfrared intense laser pulses. Phys. Rev. A.

[33] Cireasa, R. *et al*. Probing molecular chirality on a sub-femtosecond timescale, *Nat. Phys*. 10.1038/nphys3369 (2015).

[34] Suresh M (2005). Multiple ionization of ions and atoms by intense ultrafast laser pulses. Nuclear Instruments and Methods in Physics Research B.

[35] Möller M (2012). Dependence of high-order-harmonic-generation yield on driving-laser ellipticity. Phys. Rev. A.

[36] Le. AT, Lucchese RR, Tonzani S, Morishita T, Lin CD (2009). Quantitative rescattering theory for high-order harmonic generation from molecules. Phys. Rev. A.

[37] Le AT, Lucchese RR, Lin CD (2013). Quantitative rescattering theory of high-order harmonic generation for polyatomic molecules. Phys. Rev. A.

[38] Li H (2011). Orientation dependence of the ionization of CO and NO in an intense femtosecond two-color laser field. Phys. Rev. A.

[39] Holmegaard L (2010). Photoelectron angular distributions from strong-field ionization of oriented molecules. Nature Physics.

[40] Gianturco FA, Lucchese RR, Sanna N (1994). Calculation of lowâ€?energy elastic cross sections for electronâ€?CF4 scattering. J. Chem. Phys..

[41] Natalense APP, Lucchese RR (1999). Cross section and asymmetry parameter calculation for sulfur 1s photoionization of SF_6_. J. Chem. Phys..

[42] Morishita T, Le AT, Chen Z, Lin CD (2008). Accurate Retrieval of Structural Information from Laser-Induced Photoelectron and High-Order Harmonic Spectra by Few-Cycle Laser Pulses. Phys. Rev. Lett..

[43] Itatani J (2004). Tomographic imaging of molecular orbital. Nature.

[44] Baker S (2006). Probing proton dynamics in molecules on an attosecond time scale. Science.

[45] Haessler. S (2010). Attosecond imaging of molecular electronic wavepackets. Nat. Phys..

[46] Smirnova O, Mairesse Y, Patchkovskii S, Dudovich N, Villeneuve D, Corkum P (2009). High harmonic interferometry of multi-electron dynamics in molecules. Nature.

[47] Wong MCH (2013). High harmonic spectroscopy of Cooper minimum in molecules. Phys. Rev. Lett..

[48] Ni C-K, Tseng C-M, Lin M-F, Dyakov YA (2007). Photodissociation Dynamics of Small Aromatic Molecules Studied by Multi-mass Ion Imaging. J. Phys. Chem. B.

[49] Frisch MJ (2004). Gaussian 03, Revision C.02.

[50] Lewenstein M, Balcou P, Ivanov MY, L'Huillier A, Corkum PB (1994). Theory of high-harmonic generation by low-frequency laser fields. Phys. Rev. A.

[51] Keldysh LV (1965). JETP.

