# Author Correction: Femtosecond Laser Mass Spectrometry and High Harmonic Spectroscopy of Xylene Isomers

**DOI:** 10.1038/s41598-018-25623-1

**Published:** 2018-05-09

**Authors:** Abdullah Alharbi, Andrey E. Boguslavskiy, Dane Austin, Nicolas Thiré, D. Wood, P. Hawkins, Felicity McGrath, A. S. Johnson, I. Lopez-Quintas, Bruno Schmidt, Francois Légaré, J. P. Marangos, Anh-Thu Le, Ravi Bhardwaj

**Affiliations:** 1 Department of Physics, Advanced Research Complex, University of Ottawa, 25 Templeton Street, Ottawa, K1N6N5, Ontario, Canada; 2 King Abdulaziz City for Science and Technology (KACST), P.O. Box 6086, Riyadh, 11442 Saudi Arabia; 3 Blackett Laboratory, Imperial College London, London, UK; 4 Instituto de Química Física Rocasolano, IQFR-CSIC, Serrano 119, 28006 Madrid, Spain; 5 INRS-EMT, Advanced Laser Light Source, 1650 Lionel-Boulet Bvd, Varennes, J3X1S2 Canada; 6 J. R. Macdonald Laboratory, Physics Department, Kansas State University, Manhattan, Kansas 66506-2604 USA

Correction to: *Scientific Reports* 10.1038/s41598-018-22055-9, published online 28 February 2018

The original version of this Article contained a typographical error in the spelling of the author Nicolas Thiré, which was incorrectly given as Nicholas Thiré. Nicolas Thiré was also incorrectly affiliated with ‘Instituto de Química Física Rocasolano, IQFR-CSIC, Serrano 119, 28006, Madrid, Spain’. The correct affiliation is listed below.

INRS-EMT, Advanced Laser Light Source, 1650 Lionel-Boulet Bvd, Varennes, J3X1S2, Canada.

This has now been corrected in the PDF and HTML versions of the Article and in the accompanying Supplementary Information file.

