# Supplementary material for: Femtosecond Laser Mass Spectrometry and High Harmonic Spectroscopy of Xylene Isomers
Source: Sci Rep. 2018 Feb 28;8:3789. doi: 10.1038/s41598-018-22055-9 (PMC5830629; doi:10.1038/s41598-018-22055-9)
Supplement: Supplementary file 1 — supplementary information [file 41598_2018_22055_MOESM1_ESM.docx]

**Femtosecond Laser Mass Spectrometry and High Harmonic Spectroscopy of Xylene Isomers**

**Abdullah Alharbi^1,2^, Andrey Boguslavskiy^1^, Dane Austin^3^, Nicolas Thiré^5^, D. Wood ^4^, P. Hawkins^3^, Felicity McGrath^3^, A. S. Johnson^3^, I. Lopez-Quintas^4^, Bruno Schmidt^5^, Francois Légaré^5^, Jon Marangos^3^, Anh-Thu Le^6^ and Ravi Bhardwaj^1^**

^1^ Department of Physics, Advanced Research Complex, 25 Templeton Street, University of Ottawa, Ottawa, K1N6N5, Ontario, Canada

^2^ King Abdulaziz City for Science and Technology (KACST), P.O. Box 6086, Riyadh 11442, Saudi Arabia

^3^ Blackett Laboratory, Imperial College London, London, UK

^4^ Instituto de Química Física Rocasolano, IQFR-CSIC, Serrano 119, 28006 Madrid, Spain

^5^ INRS-EMT, Advanced Laser Light Source, 1650 Lionel-Boulet Blvd, Varennes, J3X1S2, Canada

^6^J. R. Macdonald Laboratory, Physics Department, Kansas State University, Manhattan, Kansas 66506-2604, USA

^*^E-mail: [ravi.bhardwaj@uottawa.ca](mailto:ravi.bhardwaj@uottawa.ca); [j.marangos@imperial.ac.uk](mailto:j.marangos@imperial.ac.uk); atle@phys.ksu.edu

Phone: +1-6135625800 Ext.6759

**1. Single ionization of xylenes with linear and circular polarized light.** Ionization of an atom or a molecule results in slightly different yields for a given intensity with the use of linear and circularly polarized light. So, to obtain similar yields for both laser polarizations, the intensity of circular polarized light has to be adjusted by a scaling factor^1^ that arises from two opposing effects. First, the electric field is always on for circularly polarized field leading to a higher cycle-average tunneling compared to linearly polarized light with the same electric field. On the other hand, the electric field is lower for circular polarization for the same value of the pulse energy by a factor of 1/√2. We measured the scaling factor in Xe to be 0.65 in good agreement with the published value^1^.

Supplementary Figure 1shows the intensity variation of the singly charged parent ion for both o- and p-xylene, obtained with linear (squares) and circular (circles) laser polarizations. The ion signal was obtained by integrating the respective *m/q* peaks. As expected, at a given intensity the yield for circular polarization is lower for both molecules compared to linear polarization. To obtain similar yields the intensity scale for circular polarized light was adjusted in Supplementary Figure 1 by a factor of 0.74 (0.68) for o-xylene (p-xylene) yield. Higher scaling factors in xylenes compared to Xe highlights the differences in tunnel ionization among different species.


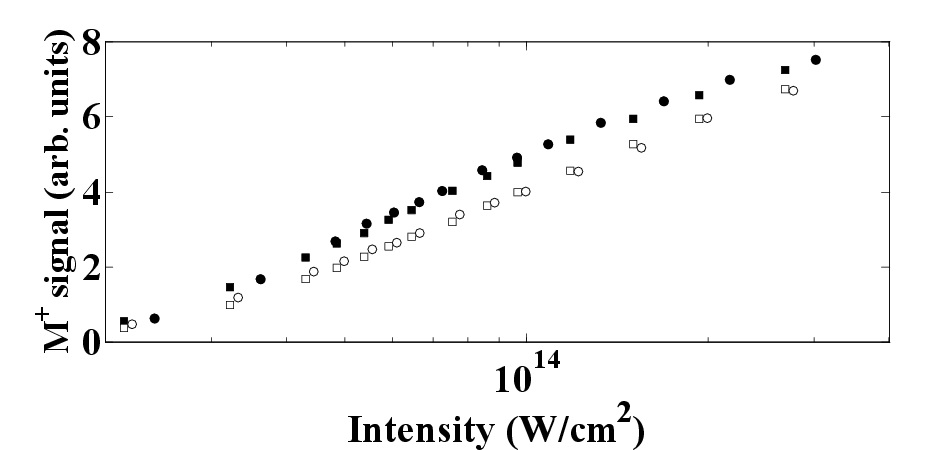
The key result of Supplementary Figure 1 is that the yield of o-xylene is slightly higher than p-xylene for both linear and circular polarizations. The result for linear polarization agrees with Urbasch *et al* work^2^**.** where only linearly polarized light was used. At a laser intensity of 1x10^14^ W/cm^2^, the ratio of the yields of p-xylene to o-xylene is 0.8, in a good agreement with a calculated ratio of 0.75. Our calculated total ionization yields (sum of HOMO and HOMO-1) were obtained by integrating emission over all directions. Results suggest the xylene two isomers exhibit differences in ionization but are small to be able to distinguish them unambiguously.

**Supplementary Figure 1.** **Ionization with linear and circular polarization.** Intensity dependence of singly charged parent ion yield at 800 nm: o-xylene (solid symbols) and p-xylene (open symbols) for linear polarization (squares) and circular polarization (circles). The intensity axis of the yields for circular polarization was shifted in both plots by a factor of 0.74 and 0.68 for o-xylene and p-xylene respectively.

**2. Double ionization of xylenes with linear and circular polarized light.** Xylene di-cations can be produced either by sequential or non-sequential ionization. In sequential ionization, multiphoton or tunneling process removes the first electron from the neutral molecule and then the second electron is removed from the ion. In non-sequential ionization, the electron removed from the neutral molecule is driven back to the parent ion by the strong laser field to undergo inelastic scattering. Depending on the energy of the recolliding electron collisional ionization or excitation occurs where the second electron is subsequently removed. While both process are present for linear polarization, recollision is completely suppressed with circular polarized light.


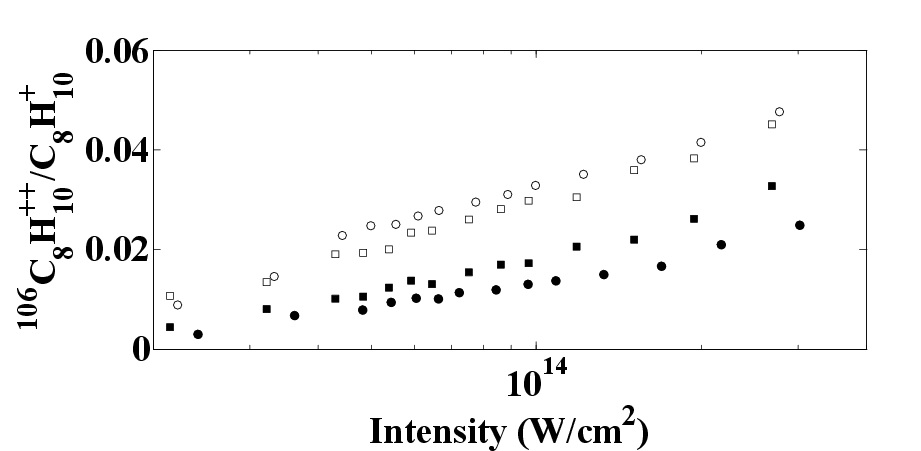
Supplementary Figure 2 shows the intensity variation of double ionization yield of o-xylene and p-xylene for linear and circular polarizations. The doubly charged ion yields were normalized to singly charged parent ion. Results suggest: (i) Di-cation yield of p-xylene is higher than o-xylene even though the singly charged ion yield of o-xylene is higher than p-xylene (Supplementary Figure 1). (ii) Xylene di-cation yields for linear polarization differ from circular polarization even after intensity scaling. Differences persist over a wide range of laser intensities. (iii) When the laser polarization is switched from linear to circular, di-cation yield of p-xylene remained unchanged or increased slightly whereas the di-cation yield of o-xylene decreased.

**Supplementary Figure 2.** **Double ionization with linear and circular polarization. a.** Intensity dependence of doubly charged parent ion yield, normalized to their respective parent ion, at 800 nm: o-xylene (solid symbols) and p-xylene (open symbols) for linear polarization (squares) and circular polarization (circles). The intensity axis of the yields for circular polarization was shifted in both plots by a factor of 0.74 and 0.68 for o-xylene and p-xylene respectively.

**3. High harmonic generation in xylenes at mid-infrared wavelengths.** Classical recollision model^3^, used to describe high harmonic generation, requires quasi-static ionization. This ionization regime can be differentiated from multiphoton ionization by the Keldysh parameter^4^ defined as $=\sqrt{\frac{I_{p}}{{2U}_{p}}}$ , where I_p_ is the ionization energy of a molecule and U_p_ ~ Iλ^2^ is the ponderomotive potential (I and λ are laser intensity and wavelength, respectively). γ ≪ 1 (γ ≫ 1) corresponds to quasi-static tunnel (multiphoton) ionization. Xylene isomers have very low ionization energy ~ 8.5 eV. As a result, at 800nm and an intensity of 5x10^13^ W/cm^2^ ionization is not quasi-static (γ ~ 1.2). At this laser intensity, longer mid-infrared wavelengths ensure ionization is adiabatic (γ ~ 0.67 at 1430nm and γ ~ 0.51 at 1850nm).

Supplementary Figure 3a,b shows high harmonic spectra of the three xylene isomers (o-, p- and m-) at two different wavelengths, 1430 nm and 1850 nm. At the shorter wavelength, the harmonic signal is lowest for m-xylene whereas the signal from p- and o-xylene is not distinct. Use of longer wavelengths enables to extend the cut-off harmonics and to differentiate the harmonic signal from p- and o-xylene.


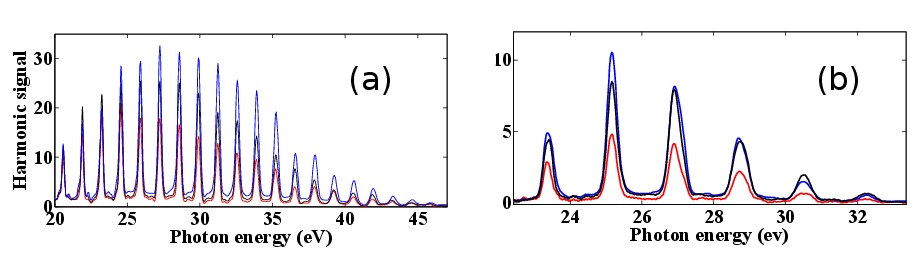
**Supplementary Figure 3. High harmonic spectra of xylenes.** HHG spectra o-xylene (blue), p-xylene (black) and m-xylene (red) obtained at **a.** an intensity of 6 x10^13^ W/cm^2^ and a wavelength of 1850 nm, and **b.** an intensity of 4x10^13^ W/cm^2^ and a wavelength of 1430 nm.

Results presented in Figure 2 of the main paper suggested o-xylene can be distinguished from p- and m-xylenes. Although the differences between p- xylene and m-xylene are small, they are measurable. These differences persist even at a shorter wavelength of 1430 nm as shown in Supplementary Figure 4 for two different laser intensities. However, at this wavelength, the ratio of o-xylene to p-xylene is close to unity making it more difficult to differentiate the two isomers.


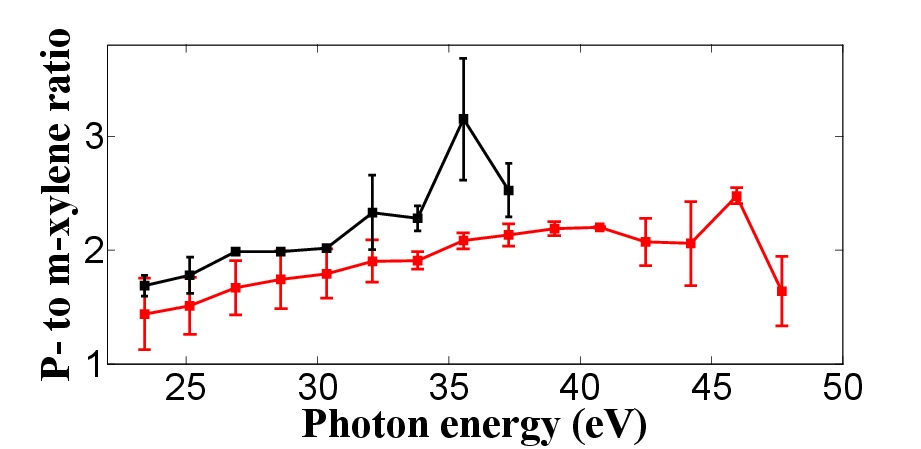


**Supplementary Figure 4. Ratio of high harmonic yields.** HHG yields ratios of p-xylene to m-xylene at 1430 nm for two laser intensities 6x10^13^ W/cm^2^ (black squares) and 1x10^14^ W/cm^2^ (red circles)

**4. Calculation of angle dependent ionization yields.** Unlike atoms, strong field ionization of molecules depends on the molecular orientation with respect to the laser field^5,6^. Ionization can be suppressed in molecules^7,8^ depending on the symmetry of the molecular orbitals due to destructive interference of electron wave packets propagating along certain directions^9^.


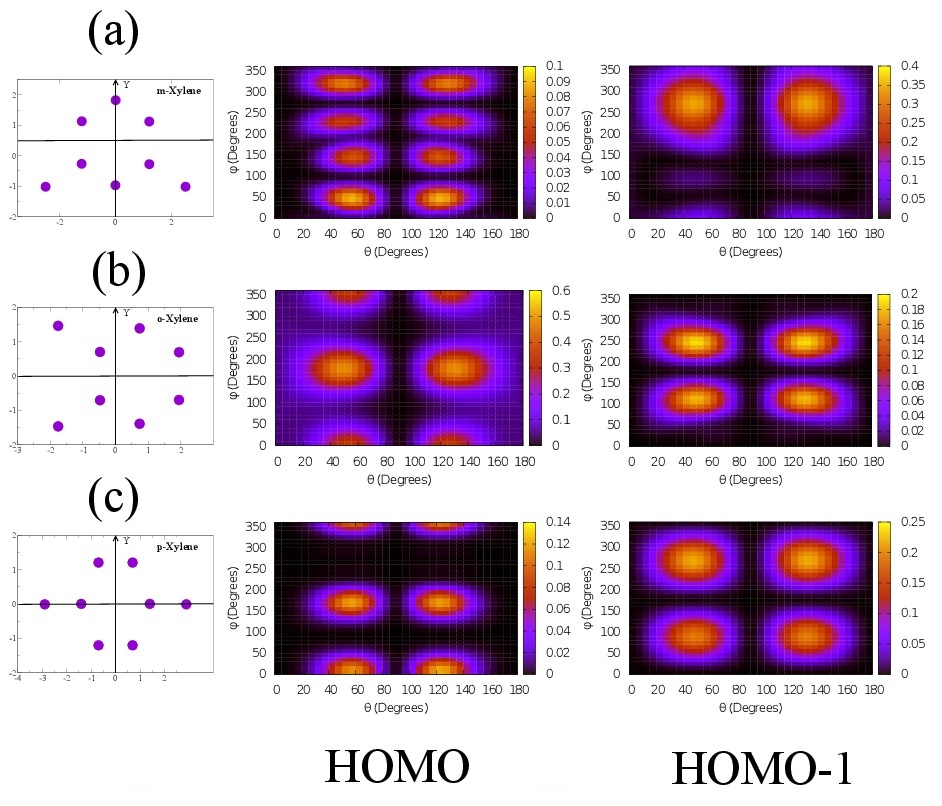
Supplementary Figure 5 shows the angle-dependent ionization yields for the three xylene isomers calculated using the Stark-corrected SFA^10,11^ for a 3-cycle pulse with the laser intensity of 0.8×10^14^W/cm^2^ and wavelength of 1850 nm. Only a half- cycle at the peak of a three-cycle pulse is allowed to ionize the molecules in order to have a definite orientation for the electric field. Only HOMO and HOMO-1 orbitals were used in our calculation since they have very close ionization energies and are well separated from other molecular orbitals. Although, the total ionization yields from HOMO and HOMO-1 for xylene isomers differ from one another only by about 20%, the angle-dependent ionization yields vary strongly.

**Supplementary Figure 5. Angle dependent ionization yields** for m-xylene (a), o-xylene (b) and p-xylene (c). The left column shows the molecular frames for the three isomers. For clarity, only carbon atoms are shown. Angle dependent ionization for HOMO is shown in the middle column and for HOMO-1 in the right column. Laser polarization direction is given by polar angle θ and azimuthal angle φ, defined in the molecular frames.

The ionization of HOMO peaks near ϑ = 50^o^ (and 130^0^) for o-xylene and at ϑ = 60^o^ (and 120^0^) for the other two isomers. For HOMO-1, the ionization peaks near ϑ = 50^o^ (and 130^0^) for all three isomer. However, the azimuthal angle at which the maximum ionization occurs is different for all three molecules. Differences in the ionization of the three isomers have significant impact on the high harmonic generation. Harmonic yield is proportional to the ionization and recombination rates. Strongest overlap between ionization and recombination occurs for o-xylene resulting in highest harmonic signal compared to others (see Figure 5 of the main paper).

**5. Ratio of xylene harmonic yields at 1430 nm.** Longer laser wavelengths ensure ionization is adiabatic, and higher order harmonics (energetic XUV photons) are generated. However, the efficiency of the HHG process decreases drastically with increasing wavelength^12^. The detrimental effects of wavelength scaling can be offset by use of higher gas pressure where phase matching is still possible^13^ but can be challenging in molecules with low vapor pressure. So, a compromise wavelength is typically in the range of 1400 – 1800 nm.

At 1430 nm, the harmonic yield of p-xylene was slightly higher than m-xylene (Supplementary figure 4). Supplementary Figure 6 shows the theoretical ratios of p- and o-xylene with respect to m-xylene at laser wavelength of 1430 nm with an intensity of 0.6 × 10^14^ W/cm^2^. The calculated ratios remain nearly the same as for 1850 nm case. However, experimental results show a stronger dependence on the laser wavelength, especially at 1430nm the ratio of o- to p-xylene is close to unity.


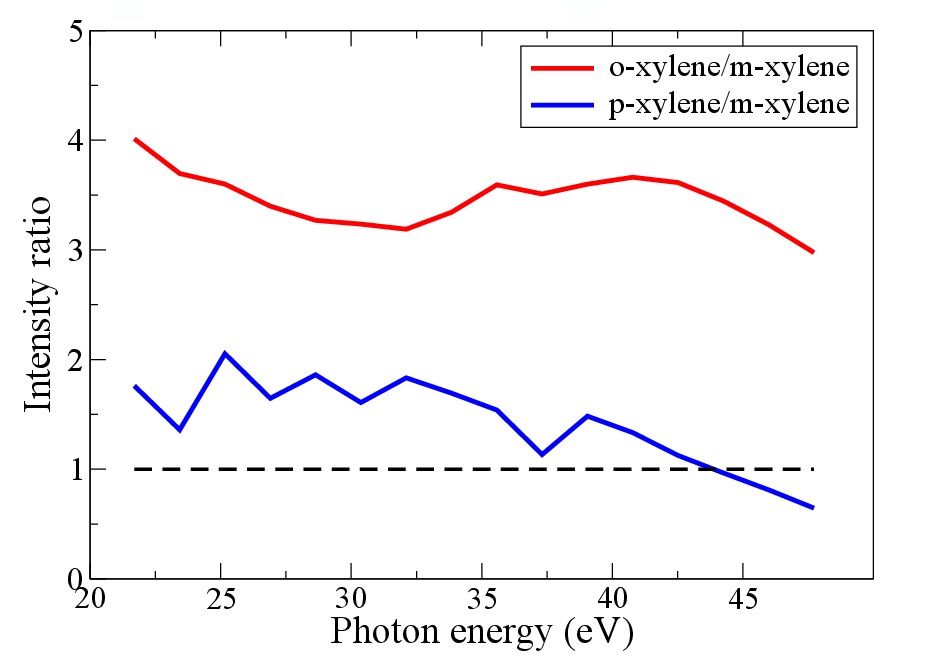


**Supplementary Figure 6. Ratio of harmonic yields.** Calculated harmonic ratios of p-xylene to m-xylene (blue) and o-xylene to m-xylene (red) at a laser wavelength 1430 nm.

**Supplementary References**

1. M. Suresh, J. McKenna, B. Srigengan, I. Williams, E. English, S. Stebbings, W. Bryan, W. Newell, E. Divall, C. Hooker, and A. Langley, Nuclear Instruments and Methods in Physics Research Section B: Beam Interactions with Materials and Atoms 235, 216 (2005)
2. G. Urbasch, H. G. Breunig, and K.-M. Weitzel, ChemPhysChem 8, 2185 (2007).
3. P. B. Corkum, Phys. Rev. Lett. 71, 1994 (1993).
4. L. V. Keldysh, Soviet Physics JETP, vol. 20, pp. 1307-1314, 1965.
5. X. M. Tong, Z. X. Zhao, and C. D. Lin, *Phys. Rev. A* **66**, 033402 (2002).
6. Lithvinyuk, I.V. et al. Alignment dependent strong field ionization of molecules, *Phys. Rev. Lett.* **90** 233003 (2003).
7. C. Guo, M. Li, J. P. Nibarger, and G. N. Gibson, *Phys. Rev. A* **58**, R4271 (1998).
8. S. M. Hankin, D. M. Villeneuve, P. B. Corkum, and D. M. Rayner, *Phys. Rev. A* **64**, 3405 (2001).
9. J. Muth-Böhm, A. Becker, and F. H. M. Faisal, *Phys. Rev. Lett*. **85**, 2280 (2000).
10. Li, H. et al. Phys. Rev. A, 84 043429 (2011).
11. Holmegaard, L., et al. Nature Physics 6 428–432 (2010).
12. Shiner, A et al, Phys. Rev. Lett. 103, 073902 (2009).
13. Popmintchev, T., et al. Opt. Lett. 33, 2128 (2008).
